# Computational Study of the Reaction between Ethylene Glycol and the CH Radical: Competition between Carbon Addition and Dehydrogenation

**DOI:** 10.1021/acs.jpca.5c06889

**Published:** 2026-02-03

**Authors:** Silvia Alessandrini, Hexu Ye, Mattia Melosso, Cristina Puzzarini

**Affiliations:** Dipartimento di Chimica “Giacomo Ciamician”, 9296 Università di Bologna, Via P. Gobetti 85, I-40129 Bologna, Italy

## Abstract

The gas-phase reaction between ethylene glycol ((CH_2_OH)_2_) and the methylidyne radical (CH) has been investigated with the aim of understanding the competition between carbon addition and dehydrogenation processes under interstellar conditions. The former type of reaction increases the molecular complexity and leads to the formation of members of the C_3_H_6_O_2_ family (with the hydrogen atom as coproduct), while the latter somewhat decreases the chemical complexity but opens the way toward the gas-phase production of C_2_H_4_O_2_ isomers (together with the CH_3_ radical as coproduct). An accurate investigation of the reactive potential energy surface indicates the formation of five isomers belonging to the C_2_H_4_O_2_ family and six species belonging to the C_3_H_6_O_2_ one. From a thermochemical point of view, the most stable product is acetic acid + CH_3_, while 2-methoxyacetaldehyde + H is the least stable. However, because of the low temperatures of the interstellar medium, reactivity is ruled by kinetics. Kinetic simulations turn the tide, with the formation of 2-methoxyacetaldehyde becoming the fastest process. The title reaction also produces glycolaldehyde + CH_3_, followed by the formation of methyl formate + CH_3_ and methyl acetate + H to a lesser extent.

## Graphical abstract

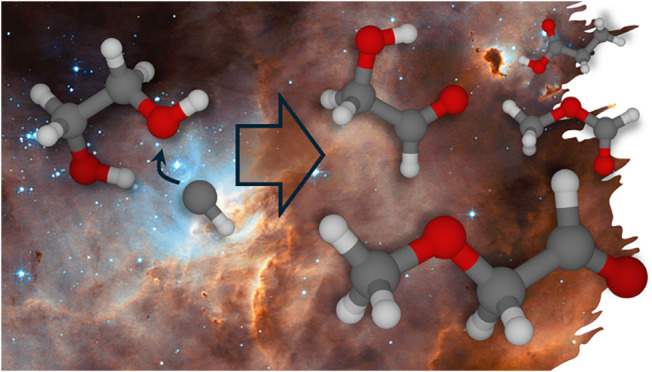

## Introduction

One of the most accredited hypotheses about the origin of life is the “RNA world” theory,^1^ which states that RNA played the dual role of processing metabolism and genetic information. Taking a step back, the exogenous delivery theory suggests an extraterrestrial role in the emergence of life: RNA precursors may have been delivered to the Earth by meteoric bombardment.^2–4^ In the framework of the “RNA world” hypothesis, sugars are crucial components. Glycolaldehyde (HOCH_2_CHO), often regarded as the simplest sugar, was first detected in interstellar medium (ISM) in 2000.^5^ The generally accepted prebiotic synthesis of sugars on early Earth is thought to occur via the formose reaction, i.e., a water-catalyzed polymerization reaction of formaldehyde (H_2_CO).^6^ However, the formation of HOCH_2_CHO on interstellar ice analogs by means of radiolysis-induced processes has also been demonstrated.^7^ In a later study, Woods et al.^8^ explored several formation mechanisms of glycolaldehyde at 10 K to assess its potential formation under typical dense core conditions. In ref 8, it was concluded that, at very low temperatures, among the possible grain-surface formation routes, only two of them −CH_3_OH + HCO and H_2_CO + HCO + H– are sufficiently efficient to produce glycolaldehyde in such an abundance to match observational estimates.

Usually, when formation mechanisms of potential prebiotic species are investigated, transformations from simple compounds to complex ones are considered. However, destruction and/or dehydrogenation processes should also be taken into account. Along these lines, this work investigates the reaction of ethylene glycol ((CH_2_OH)_2_) with the methylidyne radical (CH) to understand the possible formation of molecular species belonging to the C_2_H_4_O_2_ and C_3_H_6_O_2_ families and their connection. Members of the C_3_H_6_O_2_ family are formed in the case of the addition of the CH radical, with the H atom being the coproduct. Instead, members of the C_2_H_4_O_2_ family are produced when dehydrogenation occurs, with the CH_3_ radical being the coproduct.

The isomers of the C_2_H_4_O_2_ family are particularly relevant in astrochemistry. Indeed, in addition to glycolaldehyde mentioned above, several isomers have been detected in the ISM: methyl formate (HC­(O)­OCH_3_),^9–13^ acetic acid (CH_3_COOH),^14^ and (*Z*)-1,2-ethenediol ((CHOH)_2_).^15^ Their relative energies are shown in Figure 1: acetic acid is the most stable, while (*Z*)-1,2-ethenediol is the least one, followed when increasing the stability by glycolaldehyde and then by methyl formate. In Figure 1, 1,1-ethenediol is also reported, which lies in the energy range between methyl formate and glycolaldehyde. Therefore, this energy scale tends to suggest that 1,1-ethenediol is a promising candidate for detection in the ISM; however, the lack of the required spectroscopic characterization prevents its astronomical search.

**Figure 1:**
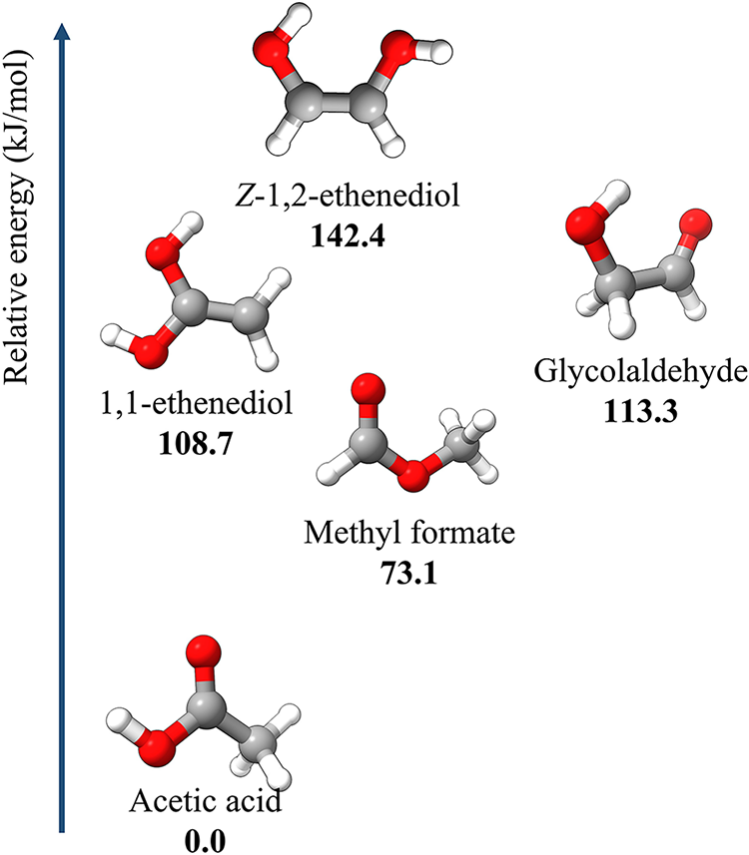
Relative energies of C_2_H_4_O_2_ isomers (in kJ·mol^–1^) at the junChS level and corrected for the harmonic ZPE contribution at the revDSD/junTZ level.

Methyl formate is rather abundant in the ISM and has been detected in both cold and hot molecular clouds (with the higher-energy trans form being recently detected^13^). While none of gas-phase reactions studied in the literature was able to explain its observed abundance, the solid-state formation of HC­(O)­OCH_3_ on CH_3_OH–CO ices irradiated with low-energy cosmic rays has been found to be the main mechanism for the synthesis of interstellar methyl formate,^16^ which is then released into the gas phase by desorption during the warm-up phase. Recently, such experimental results have been somewhat supported by atomistic calculations on water ice surface containing good CO percentages, where either CH_3_O or CH_2_OH reacts with an icy CO leading to COOCH_3_ and COCH_2_OH, respectively.^17^ In the second hydrogenation step, the former forms methyl formate, while the latter forms glycolaldehyde.

Acetic acid is also considered to be mainly produced on icy grains. It is formed whenever an ice mixture of carbon dioxide (CO_2_) and methane (CH_4_) is irradiated by high-energy electrons via the radical–radical recombination of CH_3_ and HOCO (hydroxycarbonyl radical).^7,18^ While several studies investigated the formation pathway of CH_3_COOH in interstellar ice analogs, there is still a lack of comprehensive studies on its possible gas-phase synthesis. Experiments on interstellar ice analogues composed of CH_3_OH or CH_3_OH–CO carried out by Kleimeier et al. in 2021 ^19^ pointed out the formation of (*Z*)-1,2-ethenediol. Recently, Vazart et al.^20^ and Skouteris et al.^21^ proposed a two-step reaction mechanism where the CH_2_CH_2_OH/CH_3_CHOH radicals, formed in the first step, react with atomic oxygen (O­(^3^P)) and lead to the formation of glycolaldehyde, acetic acid, and 1,2-ethenediol. However, the major products of such reactions are those resulting from the radical decomposition.

Moving to the C_3_H_6_O_2_ isomers, this family contains members that have already been found in the ISM: ethyl formate (CH_3_CH_2_OCHO),^22,23^ methyl acetate (CH_3_COOCH_3_),^23^ 1-hydroxyacetone (CH_3_COCH_2_OH)^24^ and, tentatively, 3-hydroxypropanal (HCOCH_2_CH_2_OH).^25^ Other species have been spectroscopically investigated in the laboratory and suitable line catalogs derived; however, they remain elusive in the ISM. These are propanoic acid (CH_3_­CH_2_­COOH),^26–29^ 2-hydroxy­propanal (CH_3_­CHO­HCHO),^30^ 2-methoxy­acetaldehyde (CH_3_­OCH_2_­CHO),^31,32^ 1,3-dioxolane (c-CH_2_­CH_2_­OCH_2_O),^33–36^ and glycidol (c-C_2_­H_3_O–CH_2_­OH).^37,38^ In general, this family is characterized by a large number of species that are potential interstellar candidates and show a wide variety of functional groups.

Isomerization enthalpies and the relative energies of some of the molecular species belonging to the C_3_H_6_O_2_ isomer family have been studied in refs 39,40 ; however, a comprehensive energetic investigation of the C_3_H_6_O_2_ family accounting not only for all isomers but also for their conformers has only been very recently performed in our laboratory.^100^ Our recent study confirms the energy scale previously determined. At the coupled-cluster level including up to the perturbative treatment of triples as well as the extrapolation to the complete basis set limit and core correlation effects,^41,42^ the most stable isomer is propanoic acid.^100^ The species lying within 100 kJ·mol^–1^ above the latter are, in decreasing order of stability, methyl acetate, ethyl formate, 1-hydroxyacetone, 2-hydroxypropanal, and 3-hydroxypropanal.^100^

Various synthetic routes have been proposed for the formation of hydroxyacetone and methyl acetate. These involve gas-phase ion–molecule reactions, as well as reactions on the surface of interstellar grains. A detailed overview is provided in ref 40. However, the latter paper also pointed out that the untangling of the formation routes of C_3_H_6_O_2_ isomers only scratched the surface and that none of the mechanisms proposed were verified computationally or experimentally. Most importantly, in ref 40, formation pathways for hydroxyacetone, methyl acetate, and 3-hydroxypropanal, as well as their enol tautomers were experimentally demonstrated to occur within mixed ices, analogous to interstellar ices, of methanol and acetaldehyde exposed to ionizing radiation at ultralow temperatures of 5 K.

To summarize, although several attempts have been made to explain the presence in space and abundance of the species belonging to the C_2_H_4_O_2_ and C_3_H_6_O_2_ families, a comprehensive understanding of the formation processes leading to their isomers is lacking, and to the best of our knowledge, no attempts have been made to connect these two families. This work thus aims to provide a contribution in this direction by investigating the (CH_2_OH)_2_ + CH reaction. At this point, a note on the reactants is warranted. Ethylene glycol was first detected in the ISM toward the galactic center source Sgr A* and Sgr B2 in 2002.^43,44^ Subsequently, its presence in NGC 1333-IRAS 2A, NGC 7129 FIRS2, and Orion-KL hot cores, IRAS 16293-2422, and G31.41+0.31 has been reported.^45–49^ The CH radical is instead ubiquitous in the ISM.

The manuscript is organized as follows. In the next section, the computational methodology is introduced. Subsequently, the results concerning the thermochemistry of the title reaction and its kinetics are reported and discussed. Finally, concluding remarks are provided.

## Methodology

### Reactive Potential Energy Surface

In order to explore the carbon addition and dehydrogenation processes in the (CH_2_OH)_2_ + CH reaction, a preliminary study of the reactive potential energy surface (PES) was performed at a low level of theory. In detail, calculations were carried out using the global hybrid B3LYP functional^50,51^ in conjunction with the partially augmented double-ζ jun-cc-pVDZ basis set.^52^ At this stage, all minimum structures (MINs) and the corresponding transition states (TSs) along the reaction channels were identified, with the correct connections between minima and TSs being verified by means of the intrinsic reaction coordinate (IRC) approach.^53^ In the second step, the structural and energetic characterization of each stationary point on PES was improved by resorting to the double-hybrid rev-DSDPBEP86 functional^54^ combined with the jun-cc-pVTZ basis set. Hereafter, this level of theory is denoted as revDSD/junTZ. The dispersion contributions were taken into account in all density functional theory (DFT) computations (both B3LYP and revDSD) by means of Grimme’s D3BJ model.^55,56^ In all cases, the nature of the stationary points was verified by computing the corresponding Hessian matrix.

In the last step, energetics was further improved by exploiting the junChS variant^57^ of the so-called “cheap” composite scheme.^58,59^ The starting point of this composite approach is the single-point energy calculation at the CCSD­(T)/jun-cc-pVTZ level within the frozen-core (fc) approximation, where CCSD­(T) stands for coupled-cluster singles and doubles with a perturbative treatment of triple excitations.^60^ The fc-CCSD­(T)/jun-cc-pVTZ energy is then improved by incorporating corrections due to the extrapolation to the complete basis set (CBS) limit and core–valence (CV) correlation effects. Both corrective terms were obtained using Møller–Plesset second-order theory (MP2).^61^ The CV contribution is computed as the energy difference between all-electron (ae) and fc-MP2 computations, both performed with the cc-pwCVTZ basis set.^62^ The CBS correction is obtained by applying the *n* ^–3^ formula^63^ to the fc-MP2/jun-cc-pVTZ and fc-MP2/jun-cc-pVQZ total energies. For details, interested readers are referred to ref 57.

To validate the accuracy of the junChS energies, we also carried out CCSD­(T)/CBS+CV single-point calculations for a portion of the PES. The CCSD­(T)/CBS+CV composite scheme incorporates three contributions: (i) the extrapolation to the CBS limit of the HF-SCF energy, (ii) the extrapolation to the CBS limit of the fc-CCSD­(T) correlation energy, and (iii) the CV effect contribution at the CCSD­(T) level. For the HF-SCF part, the extrapolation was carried out using Feller’s three-point formula^64^ in combination with the cc-pVTZ, cc-pVQZ, and cc-pV5Z basis sets.^65^ For the extrapolation of the fc-CCSD­(T) correlation energy, we used the two-point *n* ^–3^ formula by Helgaker and co-workers^63^ using the cc-pVTZ and cc-pVQZ sets.^65^ For the CV term, the cc-pCVTZ basis^65,66^ was employed. While the validation was based on equilibrium values, the energy values employed in the kinetic simulations are the junChS electronic energies augmented by the zero-point energy (ZPE) correction at the revDSD/junTZ level within a harmonic approximation (hZPE).

All calculations were performed using the Gaussian16 suite of programs.^67^

### Kinetic Simulations

Kinetic simulations were performed in order to derive the global rate coefficient for each multiwell reaction pathway. To this purpose, we used the MESS (master equation system solver) program^68^ for the resolution of the master equation, which integrates the rate coefficients of the elementary steps that form a given reaction channel. Global rate coefficients were computed in the 10–200 K temperature range at the low-pressure limit (10^–12^ atm). The global rate coefficients were also evaluated in the 1 – 1 × 10^–12^ atm pressure range, at a temperature of 100 K, in order to inspect whether there is a dependence on pressure. For the collisional model, Ar gas was considered as the collider and the collisional parameters were derived from ref 69, considering ethylene glycol as an alcohol. The spin–orbit coupling between the *X*_1_^2^Π_1/2_ and *X*_2_^2^Π_3/2_ states of the CH radical was also incorporated in the input of the master equation simulation using the splitting value of 26 cm^–1^ from the literature.^70^

The barrierless approach of the reactants was treated using the phase space theory (PST).^71,72^ The rate coefficient is calculated through the Gorin model,^73,74^ in which an effective *r* ^–6^ potential is expressed as

1 $$V_{\mathrm{eff}}=V\left(r_{0}\right)-\frac{C_{6}}{r^{6}}$$

where *r* is the distance between the two fragments. In the present study, the *r* coordinate was chosen as the distance between the carbon atom of the CH radical and the oxygen atom of one of the hydroxyl groups of ethylene glycol. The potential was evaluated at the revDSD/junTZ level by performing a rigid scan along *r* from 1.64 to 10 Å. In the formula above, *V*(*r*_0_) is the energy of the fragments at 10 Å, which is the distance at which the reactants are assumed not to interact. The relaxed scan confirmed the barrierless nature of the entrance channel, and the *C*_6_ parameter was obtained by fitting the revDSD/junTZ potential energies to eq 1, the derived value being 98.34 *a*_0_^6^ *E*_h_.

For each elementary step involving a TS, the unimolecular rate coefficient was computed by exploiting conventional transition state theory (TST).^75^ Specifically, the microcanonical rate coefficient was determined using the Rice–Ramsperger–Kassel–Marcus (RRKM) theory,^76,77^ which implies that the reactant is in microcanonical equilibrium. RRKM theory also required the knowledge of vibrational and rotational partition functions, the evaluation of which was based on the rigid-rotor harmonic-oscillator (RRHO) approximation. Tunneling was taken into account by resorting to the Eckart model.^78^

To model the temperature dependence of the global rate coefficients, the Arrhenius–Kooij expression was employed:^79^

2 $$k\left(T\right)=\alpha {\left(\frac{T}{300}\right)}^{\beta }\exp\left(-\frac{\gamma }{T}\right)$$

where α, β, and γ are the fitting parameters, with α representing the pre-exponential factor, β accounting for the temperature dependence of the pre-exponential factor, and γ being the effective activation energy.

## Results and Discussion

Figure 2 provides an overview of the PES for the reaction between (CH_2_OH)_2_ and the CH radical. Due to the intrinsic nature of the CH radical (which is responsible for its high energy), the PESs arising from its reactions tend to be exothermic, as pointed out—for instance—in refs 80–82 . This is also the case when CH reacts with ethylene glycol. Indeed, all of the identified channels are exothermic and characterized by submerged barriers. In Figure 2, the exothermic bimolecular products are highlighted by a red bar and also sketched in the inset. However, a better overview of their molecular structure is provided by Table 1, where their relative (with respect to the reactants) hZPE-corrected junChS energies are also reported. It is noted that Pr1 to Pr5 belong to the C_2_H_4_O_2_ family with the coproduct thus being the CH_3_ radical, while Pr6 to Pr11 are members of the C_3_H_6_O_2_ family, the H atom thus being the coproduct.

**Table 1:**
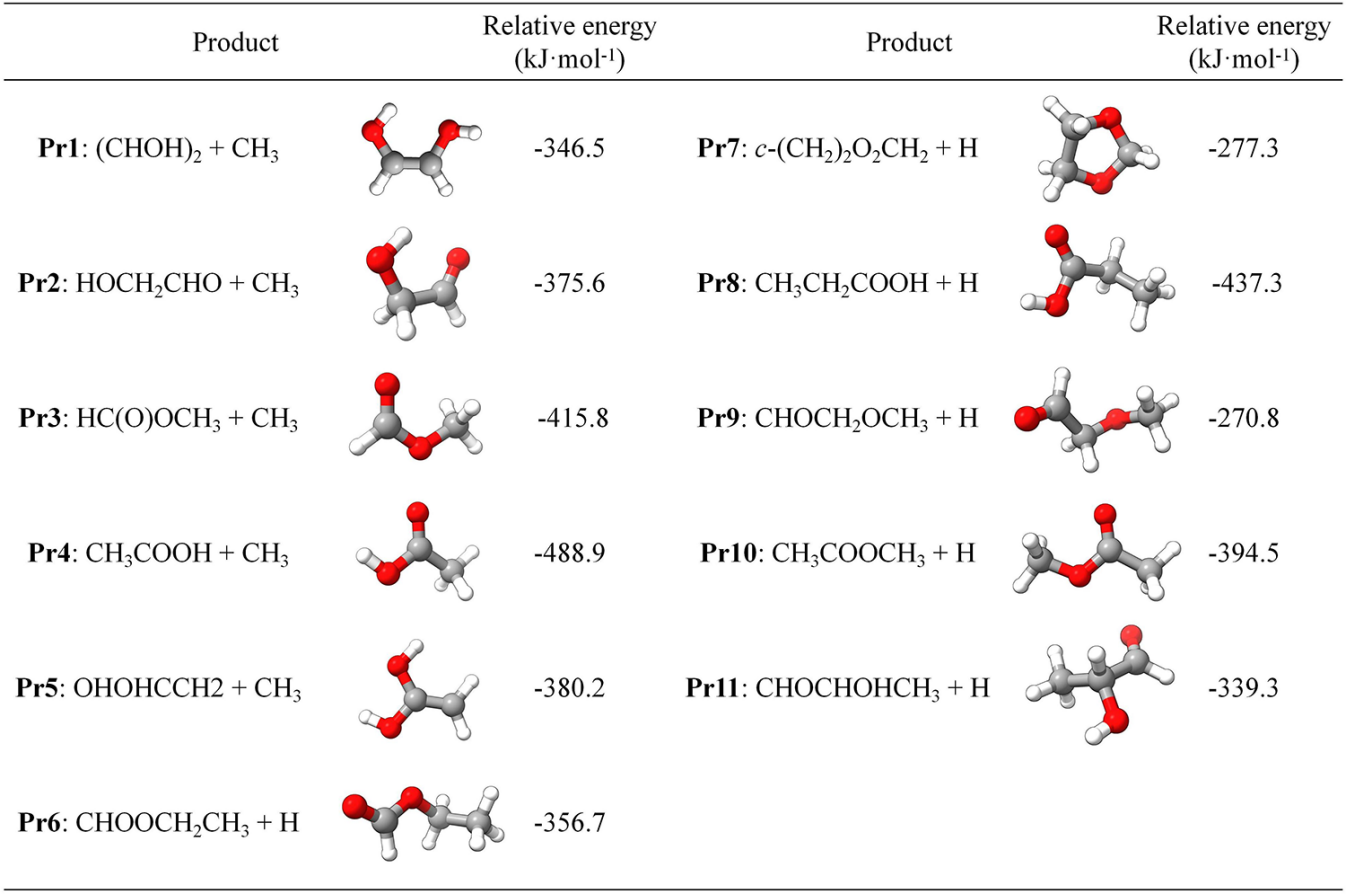
Exothermic Bimolecular Products of the (CH_2_OH)_2_ + CH Reaction^a^

a Relative energies are at the junChS level and corrected for the hZPE contribution at the revDSD/junTZ level.

**Figure 2:**
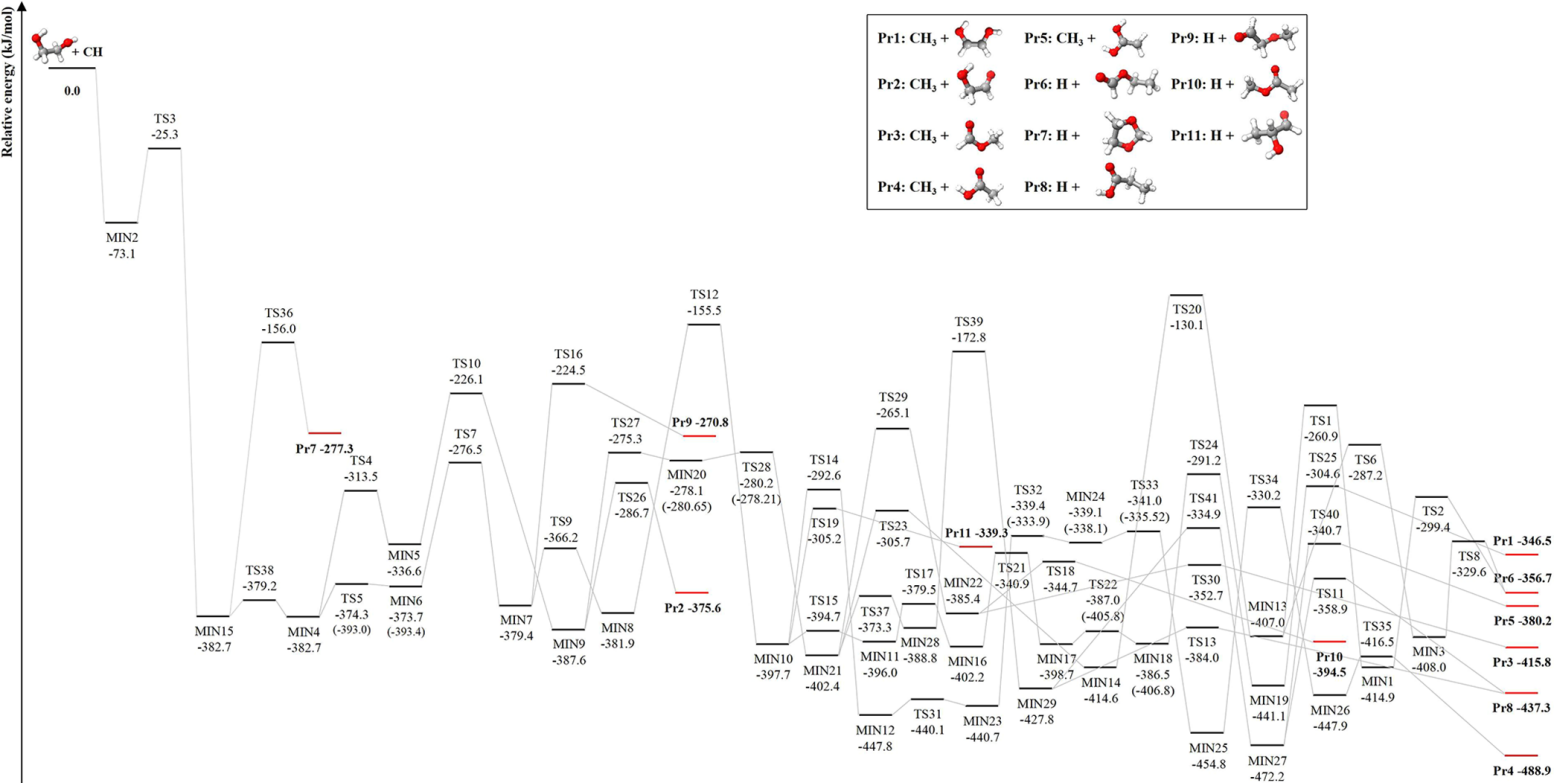
(CH_2_OH)_2_ + CH PES: junChS relative energies (in kJ·mol^–1^) incorporating harmonic ZPE corrections at the revDSD/junTZ level. In parentheses, the junChS relative energies without hZPE are reported.

The relative energies of all stationary points of the (CH_2_OH)_2_ + CH PES are not only given in Figure 2, but also summarized in Table 2, which collects the relative energies at the junChS level with and without the hZPE (revDSD/junTZ) contribution. The geometries of the MINs (intermediates) and TSs located on the PES are shown in Figures S1 and S2, respectively. Noted is that the numbering of the MINs and TSs does not reflect an energetic order, but it is instead related to the temporal evolution of the PES’s investigation.

**Table 2:** Equilibrium and hZPE-Corrected Relative Energies (in kJ·mol^–1^) of the Stationary Points of the (CH_2_OH)_2_ + CH Reaction^a^

	hZPE-corrected junChS	junChS		hZPE-corrected junChS	junChS
(CH_2_OH)_2_	–230.1640 *E*_h_	–230.2500 *E*_h_	MIN29	–427.8	–447.0
CH	–38.4658 *E*_h_	–38.4723 *E*_h_	TS1	–260.9	–267.2
reactants	0.00	0.00	TS2	–299.4	–298.8
MIN1	–414.9	–436.3	TS3	–25.3	–29.2
MIN2	–73.1	–90.6	TS4	–313.5	–324.7
MIN3	–408.0	–427.6	TS5	–374.3	–393.0
MIN4	–382.7	–403.4	TS6	–287.2	–296.8
MIN5	–336.6	–356.2	TS7	–276.5	–285.4
MIN6	–373.7	–393.4	TS8	–329.6	–331.1
MIN7	–379.4	–399.7	TS9	–366.2	–385.8
MIN8	–381.9	–402.6	TS10	–226.1	–236.0
MIN9	–387.6	–408.8	TS11	–358.9	–358.2
MIN10	–397.7	–414.0	TS12	–155.5	–171.0
MIN11	–396.0	–412.2	TS13	–384.0	–386.6
MIN12	–447.8	–468.9	TS14	–292.6	–291.0
MIN13	–407.0	–428.0	TS15	–394.7	–409.7
MIN14	–414.6	–433.5	TS16	–224.5	–221.7
MIN15	–382.7	–404.4	TS17	–379.5	–396.1
MIN16	–402.2	–421.8	TS18	–344.7	–345.5
MIN17	–398.7	–419.0	TS19	–305.2	–301.9
MIN18	–386.5	–406.8	TS20	–130.1	–138.3
MIN19	–441.1	–460.5	TS21	–340.9	–342.3
MIN20	–278.1	–287.2	TS22	–387.0	–405.8
MIN21	–402.4	–418.9	TS23	–305.7	–312.9
MIN22	–385.4	–404.1	TS24	–291.2	–300.0
MIN23	–440.7	–460.9	TS25	–304.6	–310.8
MIN24	–339.1	–348.7	TS26	–286.7	–294.0
MIN25	–454.8	–467.6	TS27	–275.3	–287.2
MIN26	–447.9	–465.3	TS28	–280.2	–291.0
MIN27	–472.2	–494.0	TS29	–265.1	–272.4
MIN28	–388.8	–405.8	TS30	–352.7	–364.4
Pr1	–346.5	–344.3	TS31	–440.1	–457.8
Pr2	–375.6	–372.5	TS32	–339.4	–350.5
Pr3	–415.8	–415.3	TS33	–341.0	–351.2
Pr4	–488.9	–487.4	TS34	–330.2	–334.8
Pr5	–380.2	–376.4	TS35	–416.5	–427.8
Pr6	–356.7	–350.9	TS36	–156.0	–162.1
Pr7	–277.3	–280.2	TS37	–373.3	–388.6
Pr8	–437.3	–432.7	TS38	–379.2	–399.3
Pr9	–270.8	–262.0	TS39	–172.8	–187.0
Pr10	–394.5	–388.0	TS40	–340.7	–346.5
Pr11	–339.3	–331.6	TS41	–334.9	–344.2

a The absolute energies (in Hartree) of the two reactants are also reported.

Focusing on the reaction mechanism, when the highly reactive CH radical approaches (CH_2_OH)_2_, it forms a carbon–oxygen covalent bond with one of the oxygen atoms of the latter molecule, thus leading to the formation of MIN2, which lies 73.1 kJ·mol^–1^ below the reactants. This step is followed by hydrogen migration via TS3 (−25.3 kJ·mol^–1^), resulting in the formation of MIN15, which has a relative energy of −382.7 kJ·mol^–1^. MIN15 then interconverts into MIN4 through an intramolecular rotation ruled by a very small energy barrier (TS38 at −379.2 kJ·mol^–1^). A subsequent hydrogen migration from the oxygen to the carbon atom produces MIN5 (−336.6 kJ·mol^–1^). Through another hydrogen transfer step, MIN9 is formed, which is located at −387.6 kJ·mol^–1^. Finally, the carbon–oxygen bond breaks, and the CH_3_ radical is released, leading to the formation of Pr2 (HOCH_2_CHO + CH_3_) via TS26 (−286.7 kJ·mol^–1^). However, going a few steps back, MIN15 is also able to directly form Pr7 (*c*-(CH_2_)_2_O_2_CH_2_ + H) by overcoming the barrier ruled by TS36 (−156.0 kJ·mol^–1^).

In addition to the formation of MIN5, from MIN4, the reaction can also proceed to MIN6 (−373.7 kJ·mol^–1^) by overcoming a very small energy barrier ruled by TS5 (−374.3 kJ·mol^–1^). When ZPE corrections are taken into account, the relative energy of TS5 is slightly lower than that of MIN6, the energy difference being, however, only 0.4 kJ·mol^–1^ (and thus smaller than the accuracy of our methodology). Subsequently, hydrogen migration between carbon atoms leads to the formation of MIN7, which has a relative energy of −379.4 kJ·mol^–1^. This evolves into MIN8 (−381.9 kJ·mol^–1^) via an energy barrier ruled by TS9 (−366.2 kJ·mol^–1^) or into Pr9 (CH_3_OCH_2_CHO + H), at −270.8 kJ·mol^–1^, by overcoming a barrier of 154.9 kJ·mol^–1^ (TS16). Back to MIN8, a hydrogen migration step proceeds through TS12 (−155.5 kJ·mol^–1^) to form MIN10, which has an energy of 397.7 kJ·mol^–1^ below that of the reactants. Starting from MIN10, the reaction pathway can evolve to Pr1 ((CH_2_OH)_2_ + CH_3_) or Pr4 (CH_3_COOH + CH_3_). The pathway toward Pr1 involves six steps: MIN10 → TS15 → MIN11 → TS17 → MIN16 → TS21 → MIN17 → TS22 → MIN18 → TS24 → MIN19 → TS25 → Pr1. Similarly, the formation of Pr4 from MIN10 also follows a six-step process: MIN10 → TS14 → MIN12 → TS31 → MIN23 → TS32 → MIN24 → TS33 → MIN25 → TS34 → MIN26 → TS35 → Pr4.

From MIN9, a path involving the migration of an OH group in a two-step process toward MIN21 (−402.4 kJ·mol^–1^) is observed: MIN9 → TS27 → MIN20 → TS28 → MIN21. Then, MIN21 is a key intermediate that opens the way toward the formation of Pr3 (CH_3_OCHO + CH_3_), Pr5 (CH_2_C­(OH)_2_ + CH_3_), Pr8 (CH_3_CH_2_COOH + H) and Pr10 (CH_3_COOCH_3_ + H), which are expected to be extremely correlated. Pr3 can be formed through a two-step process: MIN21 → TS29 (−265.1 kJ·mol^–1^) → MIN22 (−385.4 kJ·mol^–1^) → TS30 (−352.7 kJ·mol^–1^) → Pr3. Back to MIN22, Pr10 can be formed via TS18 (−344.7 kJ·mol^–1^). To form Pr8 and Pr5, MIN21 first evolves into MIN27 through the following pathway: MIN21 → TS37 (−373.3 kJ·mol^–1^) → MIN28 (−388.8 kJ·mol^–1^) → TS39 (−172.8 kJ·mol^–1^) → MIN29 (−427.8 kJ·mol^–1^) → TS41 (−334.9 kJ·mol^–1^) → MIN27 (−472.2 kJ·mol^–1^). Then, from MIN27, Pr8 is formed via TS11 (−358.9 kJ·mol^–1^), while Pr5 is formed via TS40 (−340.7 kJ·mol^–1^).

The formation of Pr6 (CH_3_CH_2_OCHO + H) also starts from MIN21, with a hydrogen migration via TS23, which lies 305.7 kJ·mol^–1^ below the reactants, leading to MIN14 (−414.6 kJ·mol^–1^) which evolves into MIN13 (−407.0 kJ·mol^–1^). From this, Pr6 can be formed via two possible routes: the first one involves path MIN13 → TS1 (−260.9 kJ·mol^–1^) → MIN1 (−419.2 kJ·mol^–1^) → TS2 (−299.4 kJ·mol^–1^) → Pr6, while the second route follows path MIN13 → TS6 (−287.2 kJ·mol^–1^) → MIN3 (−408.0 kJ·mol^–1^) → TS8 (−329.6 kJ·mol^–1^) → Pr6. The last product to consider is Pr11 (CH_3_CHOHCHO + H) that is directly formed from MIN10 via TS19, which lies 305.2 kJ·mol^–1^ below the reactants.

Before discussing the kinetic simulation results, it is important to mention the accuracy of the thermochemical data. The accuracy of the junChS scheme applied to chemical reactivity is well-demonstrated in the literature.^83–85^ Its performance has been tested with respect to the so-called HEAT-like approach,^86–90^ which is known to provide sub-kJ·mol^–1^ accuracy because it is a composite scheme entirely based on coupled-cluster theory that accounts for the CBS and CV effects at the CCSD­(T) level and incorporates corrections due to the full treatment of triple and quadruple excitations as well as relativistic and diagonal Born–Oppenheimer corrective terms. In detail, the junChS approach leads to energies that agree, within, on average, 1 kJ·mol^–1^ (with maximum deviations well within 3 kJ·mol^–1^), with those at the HEAT-like level. As mentioned in the Methodology section, a further validation has been performed in this work by exploiting the CCSD­(T)/CBS+CV composite scheme. This is shown to provide energy values with an accuracy of 2–3 kJ·mol^–1^.^86,90,91^ In detail, the portion of the PES leading to Pr2 and Pr9 has been considered and consists of the following pathways:

$$\begin{array}{l} \mathrm{Reactants}\to \mathrm{MIN}2\to \mathrm{TS}3\to \mathrm{MIN}15\to \mathrm{TS}38\to \mathrm{MIN}4\\ \mathrm{MIN}4\to \mathrm{TS}5\to \mathrm{MIN}6\to \mathrm{TS}7\to \mathrm{MIN}7\\ \to \mathrm{TS}16\to \mathrm{Pr}9\\ \mathrm{MIN}4\to \mathrm{TS}4\to \mathrm{MIN}5\to \mathrm{TS}10\to \mathrm{MIN}9\\ \to \mathrm{TS}26\to \mathrm{Pr}2 \end{array}$$

The comparison of the CCSD­(T)/CBS+CV and junChS energies, reported in Table S1, points out small differences. The mean absolute deviation is about 1 kJ·mol^–1^ and the maximum deviation observed is 3.03 kJ·mol^–1^ (for TS10). Such a result further confirms the accuracy and reliability of junChS energies, which can be confidently employed in subsequent kinetic simulations.

After the reactive PES characterization, kinetic simulations were conducted in order to understand which, among the several reactive channels, dominate and to derive the branching ratios of the different bimolecular products. Elementary and global rate coefficients have been evaluated as explained in the computational details section. Figures 3 and 4 depict the pressure and temperature behavior, respectively, of the global rate coefficients for the four fastest reaction channels, which are those leading to Pr2, Pr3, Pr9, and Pr10. However, it is evident that the production rate of 2-methoxyacetaldehyde + H (Pr9) is about 1 order of magnitude greater than that leading to glycolaldehyde + CH_3_ (Pr2) and about 3 orders of magnitude greater than those for methyl formate + CH_3_ (Pr3) and methyl acetate + H (Pr10). The rate coefficients of the other products are even by far smaller, and thus, they are not further considered. To estimate the error on the rate coefficients due to the uncertainty affecting the energy evaluation, rate coefficients have also been derived for the case in which all stationary-point energies have been increased by 5 kJ·mol^–1^ and that in which a decrease of 5 kJ·mol^–1^ has been applied; in both cases, the energies of reactants and products were kept fix at their original ZPE-corrected junChS values. The resulting rate coefficients were used to set the error bars, which are reported in Figure 4. It is observed that at low temperatures, an uncertainty of ± 5 kJ·mol^–1^ on the energetics has a negligible effect on the rate coefficients. Differently, at high temperatures, the error bars are such that an inversion in the formation rate order between methyl acetate (Pr10) and methyl formate (Pr3) might occur.

**Figure 3:**
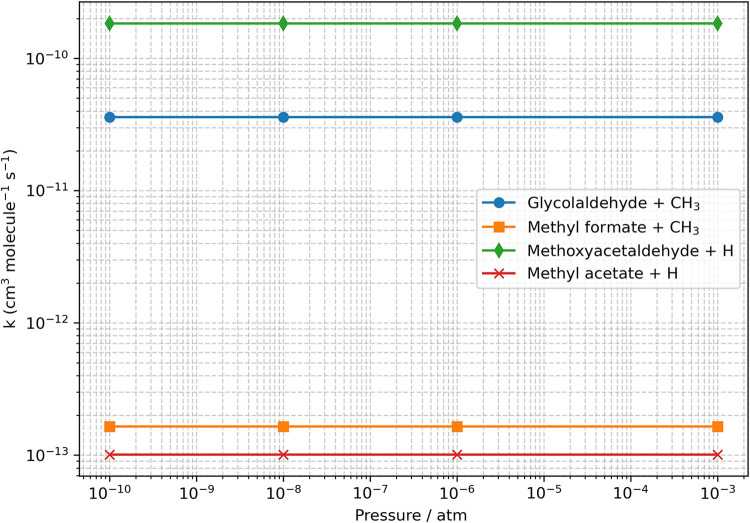
Pressure dependence of the rate coefficients (*k*), in logarithmic scale, for the formation of Pr9 (green), Pr2 (blue), Pr3 (orange), and Pr10 (red) at *T* = 100 K; hZPE-corrected junChS energies have been employed.

**Figure 4:**
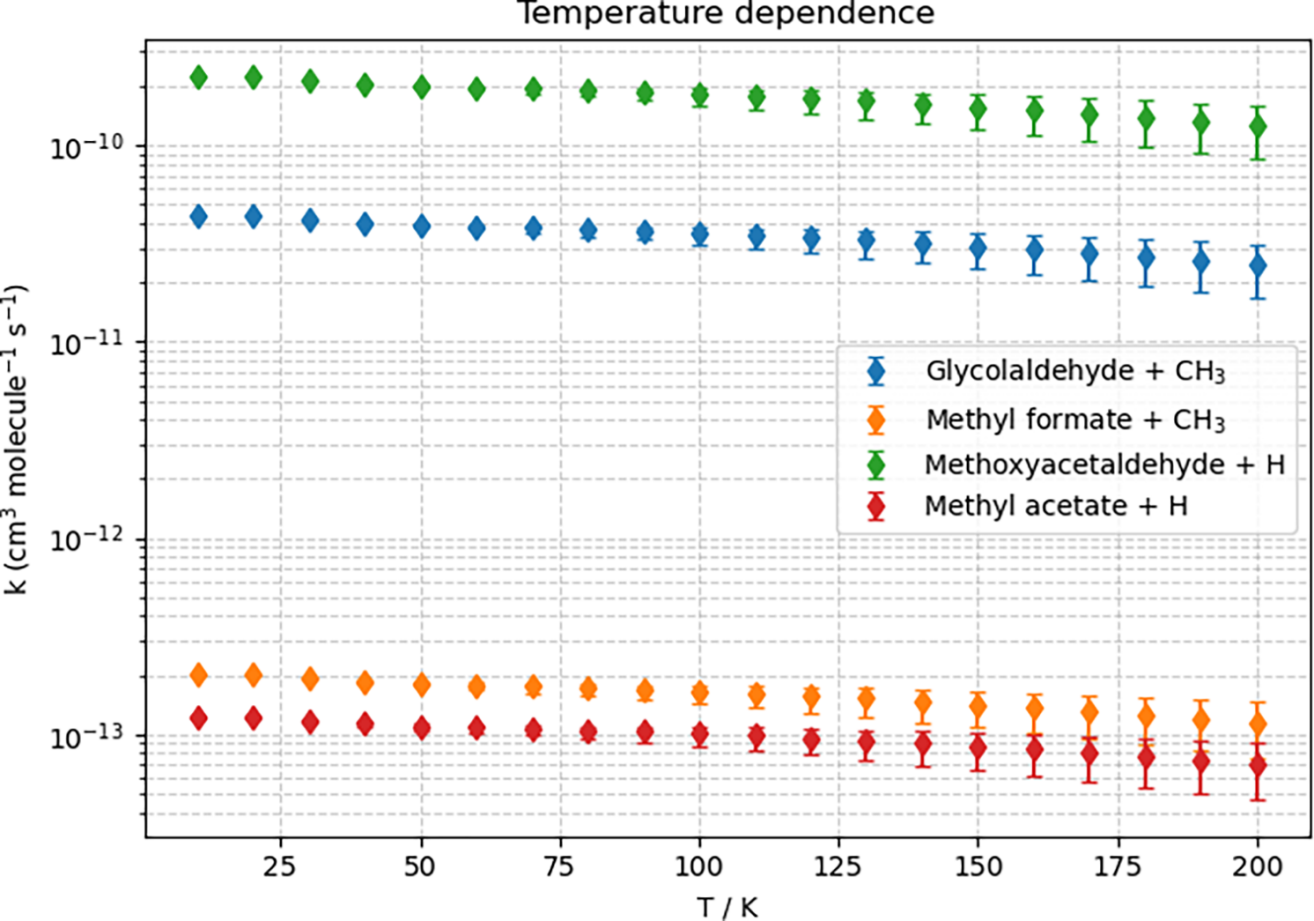
Temperature dependence of the rate coefficients (*k*), in logarithmic scale, for the formation of Pr9 (green), Pr2 (blue), Pr3 (orange), and Pr10 (red) at *p* = 10^–12^ atm; hZPE-corrected junChS energies have been employed.

The first kinetic simulations carried out concerned the pressure dependence of the global rate coefficients. As already mentioned, the 1 – 1 × 10^–12^ atm pressure range has been considered at a temperature of 100 K. In Figure 3, the behavior in the 1 × 10^–3^ to 1 × 10^–10^ atm interval is shown. It is apparent that there are no pressure effects, and therefore, the value of 10^–12^ atm can be safely assumed as the zero-pressure limit in the evaluation of the temperature dependence of rate coefficients. Moving to Figure 4, it is noted that for the four products considered, the behavior is similar: the rate coefficient decreases slightly and smoothly with increasing temperature. For Pr9, the product that is most efficiently formed, the production rate varies from 2.26 × 10^–10^ cm^3^ molecule^–1^ s^–1^ at 10 K to 1.26 × 10^–10^ cm^3^ molecule^–1^ s^–1^ at 200 K. From the global rate coefficients, the branching ratios can be derived. At 100 K, it is obtained: 83.2% for Pr9, 16.3% for Pr2, and <0.1% for Pr3 and Pr10. At 200 K, 83.5% for Pr9, 16.5% for Pr2, and <0.1% for Pr3 and Pr10. It is thus apparent that the branching ratios remain nearly unchanged within the temperature range considered. This results from the behavior of *k* upon the temperature change mentioned above, which is similar for all products. It is also evident that the title reaction significantly forms only Pr9 and Pr2, while all the remaining products are negligibly produced. The outcomes discussed above can be rationalized in an intuitive and qualitative manner by taking into consideration the highest barriers of each pathway, which can be viewed as the rate-determining steps. Therefore, Pr7 is disfavored (because of TS36) with respect to the formation of MIN4, which allows the reaction to proceed. Similarly, the bottlenecks represented by TS12 and TS29 favor the formation of Pr2 and Pr9 rather than the formation of other products (in particular Pr3 and Pr10).

The data of Figure 4 have been used to derive the Arrhenius–Kooij parameters (eq 2), which are reported in Table 3. The root-mean-square (rms) deviations demonstrate that the data are well fitted by the Arrhenius–Kooij expression. The list of the rate coefficients employed in the fit is provided in Table S2. The results of Table 3 explain why the production rates of the four channels considered show such a similar temperature dependence. Indeed, the values of β, which provide the temperature dependence of the pre-exponential factor, and the effective activation energy γ are extremely similar for all of them.

**Table 3:** Arrhenius–Kooij Parameters for the Pr9, Pr2, Pr3, and Pr10 Bimolecular Products of the (CH_2_OH)_2_ + CH Reaction

fit parameter	Pr9	Pr2	Pr3	Pr10
α, cm^–3^ molecule^–1^ s^–1^	1.30 × 10^–10^	2.54 × 10^–11^	1.18 × 10^–13^	7.21 × 10^–14^
β	–0.32	–0.32	–0.31	–0.30
γ, K	5.45	5.46	5.45	5.33
rms^a^	8.2 × 10^–12^	1.6 × 10^–12^	7.3 × 10^–15^	4.34 × 10^–15^

a rms stands for root-mean-square deviation of the fit.

## Conclusions

The present work investigates the reaction mechanisms of the gas-phase process between ethylene glycol and the methylidyne radical. As often seen for reactions involving CH, the PES is complex and shows a large number of submerged channels. Inspection of the reactive PES points out the competition between the carbon addition process, leading to the formation of six members of the C_3_H_6_O_2_ family (H being the coproduct), and dehydrogenation, which results in the production of five C_2_H_4_O_2_ isomers (CH_3_ being the coproduct). These two types of processes are strongly connected to common intermediates. To give an example, MIN27 is the last intermediate leading to both Pr5 (via TS40), belonging to the C_2_H_4_O_2_ family, and Pr8 (via TS11), a member of the C_3_H_6_O_2_ one. From a thermochemical point of view, the most exothermic product is Pr4, acetic acid + CH_3_, followed by Pr8, propanoic acid + H, which indeed correspond to the most stable isomers of the C_2_H_4_O_2_ and C_3_H_6_O_2_ families, respectively. However, at the typical low temperatures of the ISM, the most effective reaction channels are determined by the kinetic effects. Our simulations provide, at T = 100 K, a rate coefficient of 1.84 × 10^–10^ cm^3^ molecule^–1^ s^–1^ and 3.59 × 10^–11^ cm^3^ molecule^–1^ s^–1^ for Pr9 and Pr2, respectively. This corresponds to branching ratios of 83.2% and 16.3%, respectively. These values mean that under interstellar conditions, the title reaction mainly produces 2-methoxyacetaldehyde, the least stable product on the (CHOH)_2_ + CH PES, and that the addition process, which increases the chemical complexity, prevails over dehydrogenation. Since the CH radical can be easily produced by photolysis of bromoform (CHBr_3_) and reactions involving it have already been studied using the CRESU and crossed molecular-beam techniques (see, e.g., refs 92,93 and references therein), we hope that our study will encourage experimentalists to investigate the reaction between ethylene glycol and CH.

The last comment concerns astrochemical modeling. It has to be noted that, in astrochemical networks and models, ethylene glycol and glycolaldehyde are considered strongly correlated and with the same molecular origin.^94^ Therefore, we think that the results of this work provide an important piece of information to understand the correlation between these two molecules and, in general, between the C_2_H_4_O_2_ and C_3_H_6_O_2_ families of isomers. Finally, our kinetic simulations suggest that 2-methoxyacetaldehyde should be searched for in those interstellar regions where ethylene glycol is abundant.

## Supplementary Material



## Notes

Minima (Figure S1) and transition states (Figure S2) of the (CH_2_OH)_2_ + CH PES. Relative energies (in kJ·mol^–1^) at the junChS level and corrected for the hZPE contribution at the revDSD/junTZ level are also provided. Comparison between junChS and CCSD(T)/CBS+CV energies (Table S1). List of the global rate coefficients employed in the Arrhenius–Kooij fit for the channels leading to Pr9, Pr2, Pr3, and Pr10 (Table S2). (PDF)

## †

School of Materials Science and Engineering, Nankai University, Tianjin 300350, China
