# Supplementary material for: Computational Study of the Reaction between Ethylene Glycol and the CH Radical: Competition between Carbon Addition and Dehydrogenation
Source: J Phys Chem A. 2026 Feb 3;130(6):1242–52. doi: 10.1021/acs.jpca.5c06889 (PMC12908157; doi:10.1021/acs.jpca.5c06889)
Supplement: Supplementary file 1 [file jp5c06889_si_001.pdf]

Supporting information for: "Computational Study of the Reaction between Ethylene Glycol and the CH Radical: Competition between Addition and Dehydrogenation"

Silvia Alessandrini,* Hexu Ye, Mattia Melosso, and Cristina Puzzarini*

Dipartimento di Chimica "Giacomo Ciamician", Università di Bologna, Via P. Gobetti 85, 40129 Bologna, Italy

E-mail: silvia.alessandrini7@unibo.it; cristina.puzzarini@unibo.it

Table S1: Pathways of the $(CH_2OH)_2$ + CH reaction leading to Pr9 and Pr2: Comparison of the equilibrium relative energies (kJ·mol^{-1}) at the CCSD(T)/CBS+CV and junChS levels.

Species	CCSD(T)/CBS+CV	junChS	ΔE^a
Reactants (Ethylene glycol + CH)	0.00	0.0	0.0
Pr9 (Methoxyacetaldehyde + H atom)	-264.31	-264.00	0.31
Pr2 (Glycolaldehyde + CH_3)	-372.91	-372.50	0.41
TS26	-292.87	-294.00	-1.13
MIN33	-405.38	-403.40	1.98
MIN2	-90.49	-90.60	-0.11
MIN4	-404.36	-404.40	-0.04
MIN5	-357.98	-356.20	1.78
MIN6	-394.38	-393.40	0.98
MIN7	-400.83	-399.70	1.13
MIN9	-410.32	-408.80	1.52
TS10	-239.03	-236.00	3.03
TS16	-222.15	-221.70	0.45
TS3	-28.42	-29.20	-0.78
TS38	-400.18	-399.30	0.88
TS4	-326.60	-324.70	1.90
TS5	-393.95	-393.00	0.95
TS7	-285.63	-285.40	0.23

[a] Energy difference between CCSD(T)/CBS+CV and junChS.

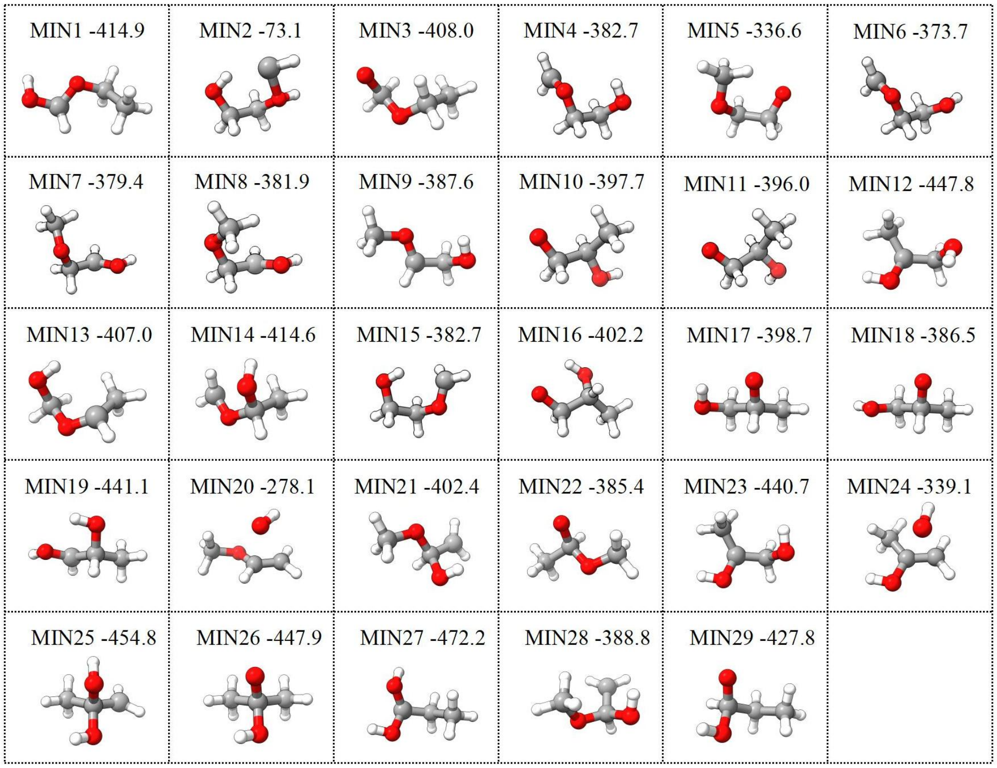

Figure S1: Minima of the $(CH_2OH)_2$ + CH PES (see Figure 2 of the manuscript). Relative energies (in kJ·mol^{-1}) are at the junChS level and corrected for the hZPE contribution at the revDSD/junTZ level.

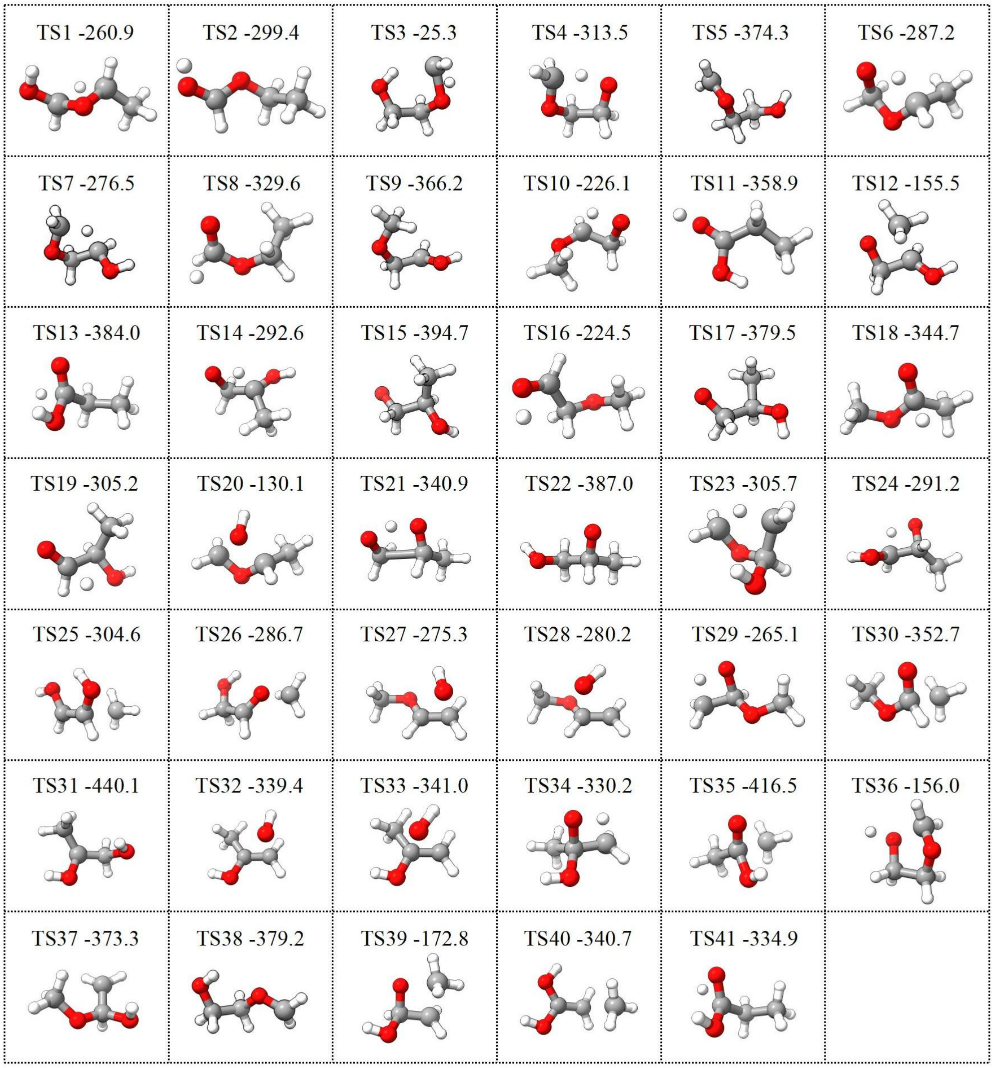

Figure S2: Transition states of the $(CH_2OH)_2$ + CH PES (see Figure 2 of the manuscript). Relative energies (in kJ·mol^{-1}) are at the junChS level and corrected for the hZPE contribution at the revDSD/junTZ level.

Table S2: Global rate coefficients (cm^3 $molecule^{-1}$) for the channels leading to Pr2, Pr3, Pr9 and Pr10 evaluated in the 10-200 K range.

Temperature	Pr2	Pr3	Pr9	Pr10
10 K	4.40E-11	2.02E-13	2.26E-10	1.22E-13
20 K	4.38E-11	2.01E-13	2.24E-10	1.22E-13
30 K	4.19E-11	1.92E-13	2.15E-10	1.17E-13
40 K	4.05E-11	1.86E-13	2.07E-10	1.13E-13
50 K	3.95E-11	1.81E-13	2.02E-10	1.10E-13
60 K	3.87E-11	1.78E-13	1.98E-10	1.08E-13
70 K	3.81E-11	1.75E-13	1.95E-10	1.06E-13
80 K	3.74E-11	1.72E-13	1.91E-10	1.05E-13
90 K	3.67E-11	1.69E-13	1.88E-10	1.03E-13
100 K	3.59E-11	1.65E-13	1.84E-10	1.01E-13
110 K	3.50E-11	1.62E-13	1.79E-10	9.85E-14
120 K	3.41E-11	1.57E-13	1.74E-10	9.59E-14
130 K	3.31E-11	1.53E-13	1.69E-10	9.31E-14
140 K	3.20E-11	1.48E-13	1.63E-10	9.01E-14
150 K	3.08E-11	1.42E-13	1.57E-10	8.70E-14
160 K	2.96E-11	1.37E-13	1.51E-10	8.37E-14
170 K	2.84E-11	1.31E-13	1.45E-10	8.04E-14
180 K	2.72E-11	1.26E-13	1.39E-10	7.70E-14
190 K	2.59E-11	1.20E-13	1.32E-10	7.35E-14
200 K	2.47E-11	1.14E-13	1.26E-10	7.01E-14
